# How can we adapt complex population health interventions for new contexts? Progressing debates and research priorities

**DOI:** 10.1136/jech-2020-214468

**Published:** 2020-09-28

**Authors:** Rhiannon Emily Evans, Graham Moore, Ani Movsisyan, Eva Rehfuess, The ADAPT Panel comprises of Laura Arnold, Julie Bishop

**Affiliations:** 1 DECIPHer, School of Social Sciences, Cardiff University, Cardiff, UK; 2 Institute for Medical Information Processing, Biometry and Epidemiology, LMU Munich, Munich, Germany; 3 Pettenkofer School of Public Health, Munich, Germany

**Keywords:** Research methods, Public health, Outcome research evaluation, Health policy, Geography

## Abstract

**Introduction:**

The UK Medical Research Council and National Institute for Health Research have funded the ADAPT study (2018–2020), to develop methodological guidance for the adaptation of complex population health interventions for new contexts. While there have been advances in frameworks, there are key theoretical and methodological debates to progress. The ADAPT study convened a panel meeting to identify and enrich these debates. This paper presents the panel’s discussions and suggests directions for future research.

**Methods:**

Sixteen researchers and one policymaker convened for a 1-day meeting in July 2019. The aim was to reflect on emerging study findings (systematic review of adaptation guidance; scoping review of case examples; and qualitative interviews with funders, journal editors, researchers and policymakers), progress theoretical and methodological debates, and consider where innovation may be required to address research gaps.

**Discussion:**

Despite the proliferation of adaptation frameworks, questions remain over the definition of basic concepts (eg, adaptation). The rationale for adaptation, which often focuses on differences between contexts, may lead to adaptation hyperactivity. Equal emphasis should be placed on similarities. Decision-making about intervention modification currently privileges the concept of ‘core components’, and work is needed to progress the use and operationalisation of ‘functional fidelity’. Language and methods must advance to ensure meaningful engagement with diverse stakeholders in adaptation processes. Further guidance is required to assess the extent of re-evaluation required in the new context. A better understanding of different theoretical perspectives, notably complex systems thinking, implementation science and realist evaluation may help in enhancing research on adaptation.

## BACKGROUND

Research on the adaptation of interventions with evidence of effectiveness for new contexts has received increased interest in population health, largely due to perceived efficiency gains over de novo intervention development. Currently, there is no consensus based guidance to support this process. In 2018, the UK Medical Research Council and National Institute for Health Research funded the ADAPT study (2018–2020).^1^ It aims to enhance the commissioning and conduct of research on intervention adaptation through development of guidance for researchers, funders, journal editors, policymakers and practitioners. The study comprises three work packages: (1) systematic review of existing guidance and scoping review of case examples; (2) qualitative interviews with stakeholders (funders, journal editors, researchers, policymakers and practitioners); and (3) Delphi exercise with stakeholders to identify best practice, consider consensus on recommendations and scope areas for further research. The first two work packages were largely undertaken during the first year of the study, in order to inform the Delphi exercise, which was conducted in year 2.

To date, the ADAPT study has identified a rapid proliferation in frameworks and guidance for prescribing intervention adaptation, largely in the fields of HIV prevention and parenting.^2^ However, the speed of this progress has meant that some fundamental and necessary theoretical and methodological debates have not been explored. In order to attend to these issues, the ADAPT study team in collaboration with the study advisory group convened a panel meeting. The need for the meeting was identified during the early phases of study delivery, where reflection on emerging work package data indicated that richer consideration of debates was required, beyond what could be feasibly explored in study team meetings. The aim was to reflect upon the findings of the ADAPT study to date; deliberate and enrich areas that lack theoretical depth or established methodological approaches; and identify research gaps that require innovation in an interdisciplinary manner. The present paper summarises the key themes from the meeting.

## Adapt study panel meeting

A panel of 16 academics and one policymaker convened for a 1-day meeting in London (July 2019). The panel comprised the ADAPT study team and the study advisory group. While representing a limited range of stakeholders, a wider set of perspectives was integrated through ongoing interviews (WP2). The agenda and discussion were structured into inter-related sections (online supplemental appendix A). ADAPT study team members provided an overview of emerging findings from the systematic review of existing adaptation guidance and stakeholder interviews, which were in progress at the time. The panel reflected on findings jointly, while attending to similarities and differences between them. Findings were further considered in relation to wider theoretical and methodological debates, and their potential relationship to existing methodological recommendations in population health.^3–5^ The meeting was audio-recorded and transcribed verbatim by a professional transcription service. Three members of the study team (GM, RE and AM) analysed the data to identify key themes.

10.1136/jech-2020-214468.supp1 Supplementary data

## Adapt study panel discussion

Five central themes emerged from the panel discussion and are considered presently. These are mapped on to questions that created significant debate, areas where there is a lack of theoretical depth or methodological approaches, and issues where the current evidence base or study findings do not offer a clear way forward.

### Theme 1: what is adaptation?

The ADAPT study was funded with a particular conceptualisation of adaptation (see definitions of key terms provided in table 1). This is the intentional modification of interventions to meet the needs of a new context, where there is an evidence-base of effectiveness in the original context. Despite this specific remit, emerging results from the ADAPT study’s findings indicate less certainty and clarity about definitions in general. To date, adaptation has largely focused on the modification of intervention components and delivery strategies. While emerging complex system perspectives have theorised ‘interventions’ as inseparable from the contexts in which they operate,^6 7^ limited attention has been paid to the need to modify aspects of the new context to accommodate the intervention. Rugged landscape theory offers interesting ways to think about why the new context may require modification, considering how the introduction of a solution to a problem cannot be understood in isolation, because its optimality depends on the solution of related problems already taking place in the landscape.^8^

**Table 1:** Key terms and definitions used in the ADAPT study

Adaptation	Intentional modification(s) of an evidence-informed intervention, in order to achieve better fit between an intervention and a new context.
Context	Any feature of the circumstances in which an intervention is implemented that may interact with the intervention to produce variation in outcomes.^9 10^
Core components	Those features in the intent and design of an intervention deemed responsible for the effectiveness of the intervention.^2^
Drift	A misapplication or a mistaken application of an intervention involving technical errors, abandonment of core components or introduction of counterproductive elements resulting in a loss of intervention benefits.^11^
Refinement	Modification(s) of an intervention to work in the same place or with the same population as originally designed and implemented.
Replication	The process of re-implementing an established intervention in a new context in a way that maintains fidelity to core goals, activities, delivery techniques, intensity and duration of the original study.^12^
Scale-out	The deliberate use of strategies to implement, test, improve and sustain an intervention as it is delivered to new populations and/or through new delivery systems that differ from those in effectiveness trials.^13^
Scale-up	The deliberate effort to broaden the delivery of an evidence-based intervention with the intention of reaching larger numbers of a target audience.^13^

The conclusion from the panel discussion was that conceptual thinking is required to differentiate adaptation from related but distinct terms. Previous research has distinguished adaptation from drift, with the latter defined as the process of unintentional or even undertheorised modifications.^11 14 15^ However, the panel recognised the difficulty in establishing the explicitness of intention or the clarity of purpose. For example, diverse stakeholders (eg, practitioners or policymakers) may modify an intervention to align with their own understanding of causal mechanisms, and so the idea of unintentional or undertheorised action is a misnomer.^16^ There is also a distinction to be made between adaptation and refinement. The panel discussed refinement as being the modification of an intervention to work in the same place or with the same population. It may be helpful to map this distinction onto the conceptual differences between scale-out and scale-up, with the former defining efforts to implement an evidence-based intervention within a new context and the latter referring to the expansion of delivery within a largely unchanged context.^13^ However, context is dynamic and further specification is needed for these definitions, in order to be more precise in how they are used.

Finally, in the ADAPT study findings to date, there has been limited consideration of whether there should be a minimal threshold of evidence in the original context for studies to be defined as cases of adaptation. Qualitative data from the ADAPT study’s interviews indicated that stakeholders variously draw upon evidence of intervention effectiveness, feasibility or acceptability to justify adaptation. Specifying criteria for the evidence-base may not be possible or helpful in practice, but it does raise important questions about the purpose of adaptation. For example, if the aim of intervention modification is to replicate effectiveness in the new context, then an outcome evaluation in both the original and new context will likely be required.

### Theme 2: when do interventions need to be adapted?

The existing literature states that interventions require adaptation primarily when there are mismatches between contexts. This is evident from the ADAPT study’s systematic review,^2^ where frameworks focus on the identification of dissimilarities as a starting point. Equally, the study’s qualitative findings indicate that the perceived uniqueness of the new context is often the driver for adaptation. Discussion among the panel reflected that there are still few decision-making approaches to assess the degree of contextual incongruence, even if there has been important progress in frameworks to map contextual features.^9 10^ There are notable challenges in making such assessments given that context is multidimensional and dynamic, with potential differences in one domain (eg, socioeconomic characteristics) and congruence in others (eg, legal frameworks). A nuanced understanding of programme theory is needed to help understand this contextual complexity. Logic models have been used alongside programme theory to depict central aspects, but while useful, they have been limited in integrating context. Rather they tend to overly emphasise linear, component-driven approaches to presenting interventions.^17^ In future, logic models should take advantage of recent progress in developing complex systems-based models,^18 19^ although there is still work to be done in operationalising how to graphically present context while maintaining usefulness. Continued work may also be undertaken to present unintended and potentially negative intervention impacts through dark logic models.

The panel also recognised a risk in focusing too much on contextual dissimilarities. It might create a culture of adaptation hyperactivity, whereby each context is considered so unique that extensive adaptation is always deemed necessary. This culture further risks neglecting de novo intervention development with potentially larger effect sizes in favour of adapting approaches with smaller impacts. Focusing on the likeness between contexts may actually reveal significant commonalities. For example, some parenting interventions have demonstrated clear transportability across contexts,^20^ perhaps because the parent–child dyad may be similar even if wider family structures are culturally distinct. As a result, frameworks to compare contexts need to be balanced, placing equal weight on the similarities and dissimilarities of relevant system characteristics. The recent TRANSFER Approach provides a useful direction, focusing on assessing the transferability of systematic review findings to the context.^21^

### Theme 3: what aspects of an intervention can be adapted and to what extent?

Even where adaptation is justified, existing research does not provide a clear consensus on what can be adapted, and at what point such extensive adaptation qualifies as de novo intervention development. Emerging findings from the ADAPT study’s systematic review indicated that frameworks draw on the concept of ‘core components’ to define the aspects of an intervention that cannot be modified.^12 22 23^ This approach reflects a common perspective held within implementation science, with an historic focus on the systematic and structured replication of evidence-based components that encase the intervention’s active ingredients.^24^ Fidelity, here is concerned with adherence to these central activities, while peripheral elements can be modified.

The panel found this dominant focus on ‘core components’ problematic. At its most simple level, discussion observed whether it was possible to disentangle core and non-core components. There were also questions about why an intervention would have activities that did not actively support the theory of change. More fundamentally, this position does little justice to the progress of complex system thinking and realist evaluation, which question the notion of interventions as a set of discrete and bounded components.^6 25^ From these perspectives, interventions are seen as system disruptions, with a clear dynamic interdependence between intervention theories, components and the system in which they operate. These interactions are notable with macro-level interventions, such as tobacco legislation. Such interventions are often simple in their components but highly complex in their processes, with impacts relying on extensive changes through the wider system (ie, society) and its subsystems (eg, tobacco industry actors).^26^ For example, introduction of smoke-free legislation across the UK from 2006 to 2007, which had high levels of compliance, may be attributed to a prolonged period of advocacy to tip the system in favour of change by securing public and political support.

These perspectives also offer a different notion of fidelity, namely functional fidelity, which was absent from the ADAPT study’s systematic review.^11 27^ This suggests that as long as the same theory of change can be activated in the new context, activities can be substituted and components adapted. Interestingly, this view resonates with the emerging findings from the ADAPT study’s qualitative data, where most stakeholders maintained that the theory of change is the key intervention element that cannot be modified. However, there is uncertainty about how to translate this approach into practice. Indeed, the panel reflected that some ideas within complex systems thinking are still based on conceptual reasoning and are difficult to operationalise due to limited empirical evidence. Therefore, methods need to be continually developed to take full advantage of all aspects of complex system perspectives, and there should be consideration of how to standardise intervention functions and adherence to them. Some examples are emerging to support this, including the recent application of ‘functional fidelity’ to re-theorise and explain mixed evidence on patient-centred medical home care.^28^

### Theme 4: who decides upon and conducts intervention adaptation?

The ADAPT study’s systematic review identified the universal importance of engaging with relevant stakeholders at multiple stages to prioritise what should be adapted.^2^ Included frameworks drew upon concepts and approaches associated with community empowerment, action research and transcreation, with the latter being defined as the development and delivery of interventions in a manner that resonates with the target population.^29 30^ More generally, there has been the application of approaches such as the Analytic Hierarchy Process to involve the target population in the cultural adaptation of interventions.^31^ The panel acknowledged the importance of this involvement, especially from a complex system perspective where the focus may be on modifying aspects of the new context in order to accommodate an intervention. However, discussion equally recognised that existing engagement processes risk being somewhat tokenistic, with the locus of power largely residing with the intervention developers. This is reflected in current nomenclature. Where modifications are undertaken by developers, often in collaboration with researchers, they are commonly defined in the literature as acts of ‘adaptation’. Even though these voices provide valuable insight into the inner workings of an intervention, they can risk allegiance bias due to a vested interest in achieving an internationally ‘branded’ product. In contrast, when other stakeholders, such as implementers or participants, modify an intervention they are seen as engaging in ‘tinkering’ or ‘drift’. Here modifications are often considered as unplanned, unintentional and misaligned with the intervention’s theory of change.

In future, the challenge within research is to enable diverse stakeholders to engage with adaptation processes without them coming from a position of relative disenfranchisement and disempowerment. It is important to achieve this while responding to the risk of intuition bias, and recognising there may be modifications that are incompatible with the intervention aims and hypothesised outcomes. To this end, there are areas where progress should be made. The language pertaining to adaptation needs to move beyond the value judgement that many stakeholders are simply tinkering. Indeed, ‘bottom-up’ adaption can make a significant contribution as it reveals how individuals respond to and act upon their immediate social system.^32^ To support this process, it is useful to draw upon learning from other research areas. For example, reverse innovation is an emerging area of population health interest, where interventions are moved from lower to higher resource settings.^33 34^ This body of work is important in troubling the dominant narrative that only certain groups, or countries, can be the driving force for innovation and change.

There is also a need to move beyond thinking of interventions as having a single and coherent theory of change. Logic models to date have compounded this thinking, and there needs to be innovative consideration of how to present multiple perspectives. In treating theory as singular, and often belonging to the intervention developer, it is easy to think that only they can adapt the intervention with intention and theoretical insight. Yet in acknowledging the multiplicity of theories in existence, it helps to understand that stakeholders may be working to enhance fidelity according to their own sense of how an intervention functions.^16^

### Theme 5: how are decisions made about re-evaluation in a new context?

The most significant evidence gap that has emerged from the ADAPT study’s systematic review^2^ and qualitative data is the paucity of decision-making approaches to determine the nature and extent of re-evaluation required in the new context. While a range of study designs are being deployed to answer questions on the replicability of intervention feasibility, acceptability and effectiveness, the rationale for their use is rarely considered. Only one framework included in the ADAPT study’s systematic review attempted to provide a conceptual frame for addressing this issue,^13^ maintaining that the degree of re-evaluation required will be contingent on the similarity of contexts and the extent of modification undertaken. Where differences are minimal, re-evaluation may focus on measures of implementation or proximal outcomes on the pathway to longer-term change. Where contexts are significantly dissimilar, and extensive modification has occurred, a replication of the outcome evaluation may be necessary.

While the panel understood this framework to be a useful departure point for considering re-evaluation, there are areas that can be further explored. First, as in the case of rationalising adaptation, there needs to be improved understanding of what it means for contexts to be similar or dissimilar. Second, it may be useful to draw more heavily upon existing frameworks for assessing the applicability and transferability of evidence.^35 36^ However, these can be limited by unrealistic criteria, requiring data not available in the new context.^35 36^ This is a pertinent issue in the field of global health, where interventions are often moved to lower resource contexts. Third, value of information (VOI) approaches can support decision-making about re-evaluation.^37^ VOI tools weigh the cost of obtaining information about an intervention (eg, effectiveness) against the value of this information in reducing uncertainty in decision-making.

## Future directions for the adapt study: development of adaptation guidance

Through discussion and progression of debates, the panel identified theoretical and methodological priorities, which are vital in moving forward research on adaptation. Centrally, there remains uncertainty and contest over the definition of key concepts, most notably adaptation itself. Rationales for intervention adaptation generally focus on differences between contexts. This may lead to adaptation hyperactivity, and frameworks for balancing both contextual differences and similarities are required. Decision-making about the types of modifications to be undertaken needs progression, taking advantage of recent efforts to operationalise constructs such as ‘functional fidelity’. Meaningful engagement with diverse stakeholders in adaptation processes should be prioritised, and more attention paid to the multiple understandings of the theories of change that may be in operation. There is also further work to be done in ascertaining the need for additional evidence in new contexts, and what types of evidence is valued.

To support this future direction, there should be more reflective and critical engagement with different theoretical perspectives to understand their implications and potential contributions.^24^ To date, implementation science seems to be the dominant perspective,^2^ with its focus on modifying intervention activities and achieving fidelity to core components. The panel discussion drew out the potential of other theoretical perspectives where relevant, notably realist evaluation and complex systems thinking. These have had minimal application to date. Yet they are highly pertinent due to their focus on the contextual contingency of effects. Realist evaluation has a strong emphasis on how proposed intervention mechanisms interact with context in generating outcomes.^38 39^ Meanwhile, complex thinking perspectives conceive intervention components as inseparable from the whole, paying close attention to the system dynamics that the intervention is intended to disrupt.^6 25–27^ The central task is to consider the possibilities of these differing perspectives, recognise their strengths and limitations, and deliberate the methods that can be used to operationalise them.

The challenge moving forward is the development and uptake of guidance to support stakeholders embarking on intervention adaptation. This includes the complexity of presenting coherent and useful recommendations, while reflecting the degree of uncertainty in a relatively recent area of research. The ADAPT study has developed a comprehensive dissemination strategy to raise awareness and encourage uptake, which includes publication, presentations and a designated website. In acknowledging the UK centric focus of the study, this activity will target a wider range of countries. Additionally, the international stakeholders participating in both the qualitative study and Delphi exercise are partly identified due to their value in developing useful and relevant guidance, and their potential to support its uptake.

Future research priorities linked to the ADAPT guidance are extensive. It is important to recognise the lack of unequivocal evidence that using adaptation frameworks, or population health guidance more broadly, necessarily leads to more effective interventions. There needs to be improved monitoring of how guidance is used and to what effect. To this end, comprehensive reporting of adaptation studies is required, similar to those for intervention description.^40^ Through the accumulation of systematic and transparently reported adaptations, there should be more clarity on what works, with recommendations for refining processes grounded in evidence. There is also the opportunity to explore the application of the guidance with different types of interventions, across disparate contexts, collating and sharing examples of its use. This can support its future refinement and expansion. Finally, as these worked examples are reported, and research on adaptation progresses, the issues considered presently should be revisited, both through empirical study and theoretical debate.

**What is already known on this subject**

- The adaptation of population health interventions for new contexts is an emerging area of research. UK Medical Research Council and National Institute for Health Research funded guidance on intervention adaptation is currently being developed.
- There are several frameworks to support adaptation processes, notably in the areas of HIV prevention and parenting.
- To date, there has been a lack of key theoretical and methodological debates in this area. This includes the potential contributions of different theoretical perspectives (eg, complex systems thinking, implementation science and realist evaluation) or the methods used to operationalise them.

**What this study adds**

- This paper progresses debates within research on adaptation, relating to definitions; rationale; the nature of modifications; stakeholder involvement; and the extent of re-evaluation required.
- Future research priorities identified include advancing the definition of key terms (eg, adaptation); progressing frameworks to assess differences and similarities between contexts; working on the operationalisation of fidelity when encouraging intervention responsiveness to local resources (eg, functionality fidelity); improving the language and methods related to stakeholder engagement; and developing decision-making tools to determine the extent of re-evaluation required in the new context.
