# Supplementary material for: How can we adapt complex population health interventions for new contexts? Progressing debates and research priorities
Source: J Epidemiol Community Health. 2020 Sep 28;75(1):40–5. doi: 10.1136/jech-2020-214468 (PMC7788480; doi:10.1136/jech-2020-214468)
Supplement: Supplementary data [file jech-2020-214468supp001.pdf]

ADAPT Innovation Meeting

Date 2/07/2019 09.00-16.30

09:00-09:30 Arrival

09:30-10:00 Introduction to ADAPT and meeting aims (RE/GM)

10:00-10:30 Update on existing MRC funded methodological guidance

- INDEX (AOC/PH)
- GUEST (LM)
- Context (PC)
- Overarching MRC framework (LM)

10:30-10:45 Reflections on synergies between ADAPT and other guidance (GM)

10:45-11:00 BREAK

11:00-11:30 Update on Work Packages 1 / 2 and Summary of Key Themes (AM/LC/HL)

11:30-12:00 Part 1: Group discussion on priority areas for innovation, organised around WP 1 / 2 (All)

1. Concepts and definitions
2. Pre-requisites for adaptation
3. Types of adaptation

12:00-13:00 LUNCH

13:00-13:20 Part 2: Group discussion on priority areas for innovation, organised around WP 1 / 2 (All)

4. The adaptation process
5. Evaluating adaptations

13:20-14:00 Summary and priority areas for innovation (RE)

14:00-14:15 BREAK

14:15-14:45 Reflections on consensus processes in INDEX and GUEST (and the role of consensus in guidance development) (All)

14:45-15.30 Planning for ADAPT DELPHI (PC)

15.30-16:00 Drafting and consulting on ADAPT guidance

16:00-16:15 Close
